# A Case of Pediatric Myasthenia Revealed by Marked Eyelid Ptosis After Using Cyclopentolate Eye Drops

**DOI:** 10.7759/cureus.79945

**Published:** 2025-03-03

**Authors:** Kaori Komatsu, Tomoko Yoshikawa, Yoshiaki Kiuchi

**Affiliations:** 1 Ophthalmology and Visual Science, Hiroshima University, Hiroshima, JPN

**Keywords:** acetylcholine receptor antibodies, cyclopentolate hydrochloride, neuromuscular etiology, pediatric myasthenia gravis, ptosis

## Abstract

Pediatric myasthenia gravis (PMG), while uncommon, poses significant diagnostic challenges due to its potential impact on development and its often subtle initial symptoms, such as ptosis and diplopia. This case report details an unusual presentation of PMG in a three-year-old female, initially diagnosed with a chalazion, highlighting the complexity of diagnosing this autoimmune neuromuscular disorder in children. Despite treatment for the assumed chalazion, the child's ptosis persisted, and disparities in visual acuity between her eyes became evident. The turning point in her diagnosis came during a routine ophthalmologic assessment using cyclopentolate hydrochloride, which exacerbated her ptosis, prompting further neuromuscular investigation. This led to the detection of acetylcholine receptor antibodies and a suspected diagnosis of PMG after a repetitive nerve stimulation test. This case underscores the critical role of detailed clinical observation and the consideration of neuromuscular etiologies in children presenting with ocular symptoms. It also emphasizes the importance of integrating the observations of family members and caregivers into clinical assessments. Oftentimes, subtle symptoms noted by those close to the patient can provide crucial clues in diagnosing complex conditions like PMG. Early detection is paramount to prevent complications such as amblyopia and to initiate timely interventions, as demonstrated by the subsequent implementation of an eye patch to preserve visual function in the affected eye of the patient. This report contributes to the growing literature on the variable presentations of PMG and underlines the necessity for vigilance and a comprehensive approach in pediatric ophthalmologic evaluations to ensure that significant underlying conditions are not overlooked.

## Introduction

Myasthenia gravis (MG) is an autoimmune neuromuscular disorder characterized by fluctuating muscle weakness, commonly worsening with activity [1,2]. This condition can be particularly challenging to diagnose in children, where it might mimic other neurological disorders, making vigilance and comprehensive assessment crucial [3,4].

Pediatric myasthenia gravis (PMG) is less common but significant due to its impact on development [5,6]. It often presents with ocular symptoms like ptosis and diplopia, which may be initially subtle but can evolve into more generalized muscle weakness [7,8]. Ptosis is worse during the day and after exercise, and systemic symptoms include dysphagia, developmental problems, and muscle weakness in the extremities [3]. Diagnosis in children hinges on clinical assessments, serological tests for acetylcholine receptor antibodies, and electrophysiological studies [9-11].

This case report describes an atypical presentation of PMG in a child. Routine ophthalmologic evaluation with the muscarinic antagonist cyclopentolate hydrochloride unexpectedly led to an exacerbation of ptosis and subsequent diagnosis of MG. This case highlights the fact that simple ophthalmic manifestations may signal a serious health problem and the importance of listening carefully to the observations and concerns of family members and those who interact with the patient daily when examining pediatric patients.

## Case presentation

The patient in this case is a three-year-old female whose right eyelid ptosis was noticed by a family member at approximately three years and six months of age. A local physician initially diagnosed the child with a right upper eyelid chalazion and prescribed betamethasone sodium phosphate and framycetin sulfate eye drops three times a day. The chalazion shrank but the droopy eyelid persisted, and a subtle difference in visual acuity was noted between the two eyes. Because of these persistent symptoms, the patient was referred to the Department of Ophthalmology, Hiroshima University Hospital, at the age of three years and 10 months.

**Figure 1:**
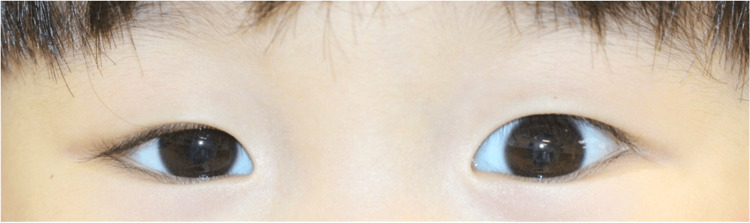
Observations of the eye at the time of initial examination (age three years and 10 months) Eye position was positive in both near and far vision. In the anterior region, the margin reflex distance (MRD)-1 was 1.5 mm in the right eye and 3.0 mm in the left eye. In addition, there was evidence of a chalazion on the nasal side of the upper eyelid conjunctiva.

On examination, visual acuity was 1.0 in both eyes as measured by the Morizane Dot Card Test for Visual Acuity, and single optotype visual acuity was 0.5 in the left eye and 0.6 in the right eye. Eye position was normal in both near and distance vision, eye movements were unrestricted, and convergence was adequate (Fig. 1). Anterior segment examination revealed residual mild traces of chalazion on the nasal aspect of the right upper eyelid (Fig. 1).

**Figure 2:**
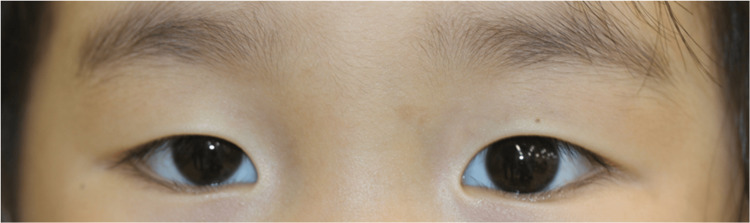
Observations of the eye 10 months after the initial examination (four years and eight months) Although there was no recurrence of chalazion, there was a difference in visual acuity between right and left eye, so a refractive examination under cyclopentolate hydrochloride eye drops was scheduled at the next visit.

The child's family reported no diurnal fluctuations in eyelid position or visual symptoms. Therefore, all medications were temporarily discontinued, and the patient was monitored; 10 months later, at age four years and eight months, the ptosis remained, and the visual acuity difference was more pronounced; the visual acuity in the right eye was 0.6, and that in the left eye was 1.0. Therefore, a refractive examination was planned (Fig. 2).

**Figure 3:**
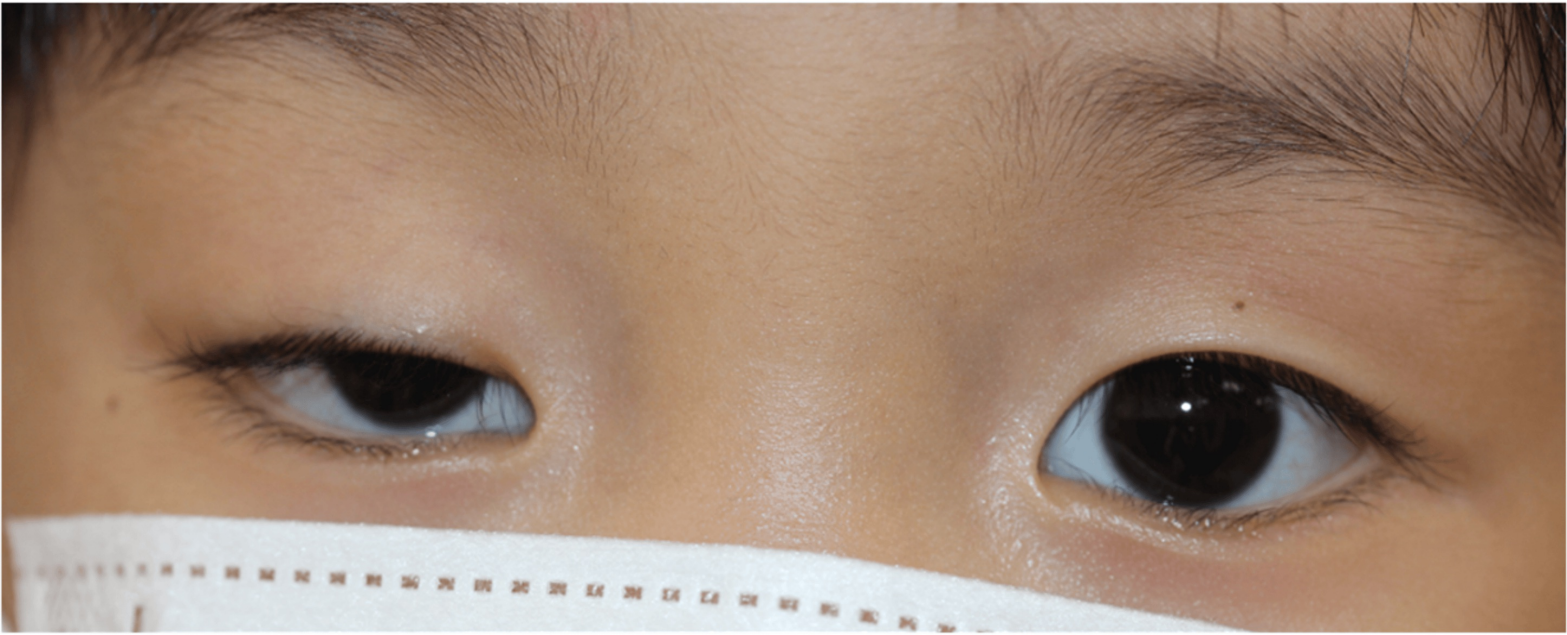
Observations in the eye one hour after cyclopentolate hydrochloride ophthalmic drops An examination conducted one hour later revealed a significant worsening of the right ptosis (MRD-1 of -1.0 mm). The refraction examination further revealed mild hyperopia in the right eye and moderate hyperopia in the left eye.

Cyclopentolate hydrochloride was administered, and an examination one hour later revealed a marked worsening of the right ptosis (MRD-1 of -1.0 mm), but the left eyelid was unchanged (Fig. 3). Refraction examination after cyclopentolate revealed mild hyperopia in the right eye and moderate hyperopia in the left eye (Fig. 3).

Neuromuscular causes were a concern, as the patient had significant ptosis after cyclopentolate hydrochloride administration. The patient was referred to the pediatric department for a thorough systemic evaluation. Blood work revealed acetylcholine receptor antibodies. A repetitive nerve stimulation test revealed a diagnosis of suspected subclinical generalized MG.

As a result of these findings, the ophthalmology team initiated a left eye patch to prevent amblyopia, given the risk of ptosis inhibiting visual input. This intervention was intended as a precautionary measure against potential problems with visual acuity development. Because there were no systemic abnormalities other than in the ocular region and her vision improved with the eye patch, the patient did not receive any other treatment, such as steroids, and was followed up.

## Discussion

This case highlights two points. First, it underscores that simple eye problems in children, such as ptosis, may signal a more serious health problem, such as PMG. Furthermore, this case emphasizes the need to listen carefully to the complaints of those who are in daily contact with the patient, such as family members, when examining children with diseases.

The first importance of recognizing that seemingly simple pediatric ocular symptoms such as ptosis may indicate a more serious underlying condition, including PMG. In this case, the patient's mild ptosis was initially thought to be a benign ophthalmologic problem but was later found to be a manifestation of PMG due to exacerbation after cyclopentolate hydrochloride administration. Previous studies have shown that PMG is characterized by the appearance of easy fatigability [5]. The reason for the development of ptosis in this case may have been the appearance of easy fatigability due to the time required for the entire examination, as the patient had to wait one hour after the two eye drops were administered. Moreover, prior studies have shown that early detection and treatment are important for PMG patients to achieve early remission [5]. Moreover, if the ptosis is left untreated, complications such as amblyopia may develop [12]. Therefore, early detection and diagnosis are extremely important to prevent various serious diseases, including PMG. Considering the above discussion, ophthalmologists and pediatricians should examine patients with seemingly simple eye symptoms, keeping in mind their association with other underlying diseases, so that serious diseases are not overlooked.

The second important aspect is for those who routinely interact with pediatric patients, especially family members, to consider their observations and concerns when evaluating a child for potential health problems. In this case, the family first noticed the child's droopy eyelids, which were eventually diagnosed as PMG. Family perspectives and information are essential for quality clinical decision-making when seeing children who are not good at explaining their own symptoms [13,14]. Thus, adequate communication with caregivers is an important component of the clinical evaluation of pediatric conditions, especially in the diagnosis of complex conditions such as PMG. One previous study identified social support, mutual respect, trust, active listening, interpersonal relationships, nonverbal cues, empathy, and confidentiality as important elements in the interview [15]. The above discussion highlights the importance of listening to the complaints and concerns of family members and others when examining children for illnesses, and in doing so, it is necessary to listen with attention to several points.

## Conclusions

This case report underscores that seemingly minor ocular problems in children, such as ptosis, may represent a more serious condition, such as PMG. In addition, this case report also underscores the importance of carefully considering the observations and concerns of family members and others who interact with the patient daily when evaluating pediatric cases. In the future, we believe that collecting more cases to clarify the symptoms of PMG and to examine strategies for interviewing family members and others will contribute to the early detection and diagnosis of PMG.

## References

[1] Cejvanovic S, Vissing J (2014). Muscle strength in myasthenia gravis. Acta Neurol Scand.

[2] Behin A, Le Panse R (2018). New pathways and therapeutic targets in autoimmune myasthenia gravis. J Neuromuscul Dis.

[3] Della Marina A, Trippe H, Lutz S, Schara U (2014). Juvenile myasthenia gravis: recommendations for diagnostic approaches and treatment. Neuropediatrics.

[4] Gilhus NE (2016). Myasthenia gravis. N Engl J Med.

[5] Finnis MF, Jayawant S (2011). Juvenile myasthenia gravis: a paediatric perspective. Autoimmune Dis.

[6] Wang H, Su Z, Luo C (2013). The effect of steroid treatment and thymectomy on bone age and height development in juvenile myasthenia gravis. Neurol Sci.

[7] Shuey NH (2022). Ocular myasthenia gravis: a review and practical guide for clinicians. Clin Exp Optom.

[8] Jayam Trouth A, Dabi A, Solieman N, Kurukumbi M, Kalyanam J (2012). Myasthenia gravis: a review. Autoimmune Dis.

[9] Peragallo JH (2017). Pediatric myasthenia gravis. Semin Pediatr Neurol.

[10] Vincent A, Newsom-Davis J (1985). Acetylcholine receptor antibody as a diagnostic test for myasthenia gravis: results in 153 validated cases and 2967 diagnostic assays. J Neurol Neurosurg Psychiatry.

[11] Kroczka S, Stasiak K, Kacinski M (2016). Neurophysiological parameters in myasthena gravis in children in diagnostic and therapeutic view [Article in Polish]. Przegl Lek.

[12] Finsterer J (2003). Ptosis: causes, presentation, and management. Aesthetic Plast Surg.

[13] (2012). Patient- and family-centered care and the pediatrician's role. Pediatrics.

[14] Vatne TM, Slaugther L, Ruland CM (2010). How children with cancer communicate and think about symptoms. J Pediatr Oncol Nurs.

[15] Khawaja M (2021). Using appreciative inquiry to explore effective medical interviews. Behav Sci (Basel).

